# Application of Mendelian randomized research method in oncology research: bibliometric analysis

**DOI:** 10.3389/fonc.2024.1424812

**Published:** 2024-12-17

**Authors:** Jiahao Chen, Yunli Wang, Rongsheng Jiang, Yawei Qu, Yan Li, Yang Zhang

**Affiliations:** ^1^ College of Medical Information, Changchun University of Chinese Medicine, Changchun, China; ^2^ College of Acupuncture and Tuina, Changchun University of Chinese Medicine, Changchun, China

**Keywords:** cancer, Mendelian randomization, bibliometric analysis, epidemiology, cancer risk, tumors, carcinoma, genome-wide association studies

## Abstract

**Background:**

Cancer has always been a difficult problem in the medical field, and with the gradual deepening of Genome-wide association studies (GWAS), Mendelian randomization methods have been increasingly used to study cancer pathogenesis. In this study, we examine the literature on Mendelian cancer, summarize the status of the research, and analyze the development trends in the field.

**Methods:**

Publications on “Mendelian Randomization - Cancer” were retrieved and downloaded from the Web of Science Core Collection database. CiteSpace 6.2.R4, VOSviewer 1.6.19, Scimago Graphica 1.0.38, Bibliometrix R-package, and a bibliometric online analysis platform were used for data analysis and visualization. An in-depth analysis of country or region, authors, journals, keywords, and references was performed to provide insights into the content related to the field.

**Results:**

A total of 836 articles were included in the analysis; 643 authors from 72 countries had published articles related to the field. China and Harvard University (among countries and institutions, respectively) had the highest number of articles. Martin, Richard M and Smith, George Davey were the largest contributors. A total of 27 cancers have been studied, with breast, colorectal, and liver cancers being the most studied.

**Conclusion:**

This study is the first to use bibliometric methods to visualize the application of Mendelian randomization analysis in the field of cancer, revealing research trends and research frontiers in the field. This information will provide a strong reference for cancer researchers and epidemiologic researchers.

## 1 Introduction

Cancer is caused by excessive proliferation of cells in the body after losing normal regulation (1). When an organism is affected by chemical, physical, viral, and other carcinogenic substances in the environment or by its own genetic, endocrine, sex, age, and other factors, a series of genetic abnormalities and changes can occur, resulting in the formation of malignant tumors. In the early stages of cancer, there are mostly no obvious symptoms, and it is difficult to detect abnormalities on a general physical examination. However, when the tumor is large or invasive, metastasis occurs and symptoms appear; thus, most patients enter the advanced stages of cancer (2, 3).

Cancer prevention and treatment remain a difficult problem that perplexes the medical community. The complex regulatory mechanisms in the body limit research on cancer pathogenesis. In recent years, Mendelian randomization has been widely used in cancer research owing to its unique advantages.

Mendelian randomization is a statistical method based on genetic variation. The core idea of Mendelian randomization is to study the causal relationship between “exposure factors” and “outcome variables” (4–6). In the process of designing experiments, single nucleotide polymorphisms (SNP) in Genome-wide association studies (GWAS) are often utilized as “instrumental variables” (7), which are ultimately utilized to reveal the causal relationship between the “exposure factor” and “outcome variable,” and to exclude the “confounding factors” associated with the “outcome variable”. Compared with traditional epidemiological research methods, Mendelian randomization can effectively eliminate confounding factors and reverse-proof causality, which avoids bias in the results caused by confounding factors (8). Genome-wide association studies (GWAS) of cancer are becoming increasingly popular in the field of epidemiology. So far, more than 70 cancer susceptibility genes (CSGs) have been identified, which can be effectively identified by comparing the frequency of DNA variation with that of healthy individuals (9). GWAS-based Mendelian randomization studies have constituted a significant advancement in the field of epidemiology, and are of paramount importance in the effort to alleviate the global burden of disease.

Bibliometric analysis is a popular disciplinary analysis method based on applied mathematics and statistics and has been widely used in the research of various disciplinary fields (10). The core idea of bibliometric analysis is to collect metadata (e.g., keywords, references, journals, abstracts) from relevant literature and perform statistical analysis of the data so that the hidden correlations between the data can be identified. The results can be further elaborated with the help of visualization tools, which make it convenient for researchers to have a more comprehensive understanding of the discipline or field (11, 12).

We conducted a bibliometric analysis of publications that used Mendelian randomization methods to study cancer over the period 2003-2024 to understand the research trends in the field of cancer over the last 20 years. We summarize the current state of research and research hotspots in the field and analyze the trends in the field in detail.

## 2 Materials and methods

### 2.1 Data sources and search strategies

To ensure the authority of the original file, data were retrieved and downloaded from the Web of Science Core Collection (WoSCC) (Index: Science Citation Index extension [SCI-E]). To further study the latest development trends in this field, the time limit was from January 2003 to January 2024, during which the Mendelian Randomization (MR) experiment was widely used in the field of cancer research. To facilitate the statistical analysis of the literature data, we only included English literature. The search was limited to Web of Science (WOS) database subject terms. The following keywords were used: TS= (Mendelian randomization Study AND Mendelian randomization) AND TS= (cancer).

### 2.2 Inclusion and exclusion criteria

It was necessary to screen the retrieved literature to ensure the reliability of the data used for the analysis. Two authors independently reviewed the documents in accordance with the following criteria, and any differences were resolved through consultation with third parties.

Inclusion criteria (1): the research topic of the article involves Mendelian randomization Study and cancer; (2) the document type is “article”; (3) the document language is limited to “English”; (4) the publication time is from January 1, 2003, to January 31, 2024.

Exclusion criteria: (1) the topic of the study is not Mendelian randomization and cancer; (2) the document type is “review articles”; (3) withdrawn or duplicate publications; (4) documents that cannot provide the basic information required for bibliometric analysis.

Each record contained the information required for data analysis, such as title, author, keywords, abstract, time, journal information, references, and country. As the data were retrieved from open databases, there were no ethical issues related to access for this study.

### 2.3 Data analysis and graph acquisition

**Figure 1:**
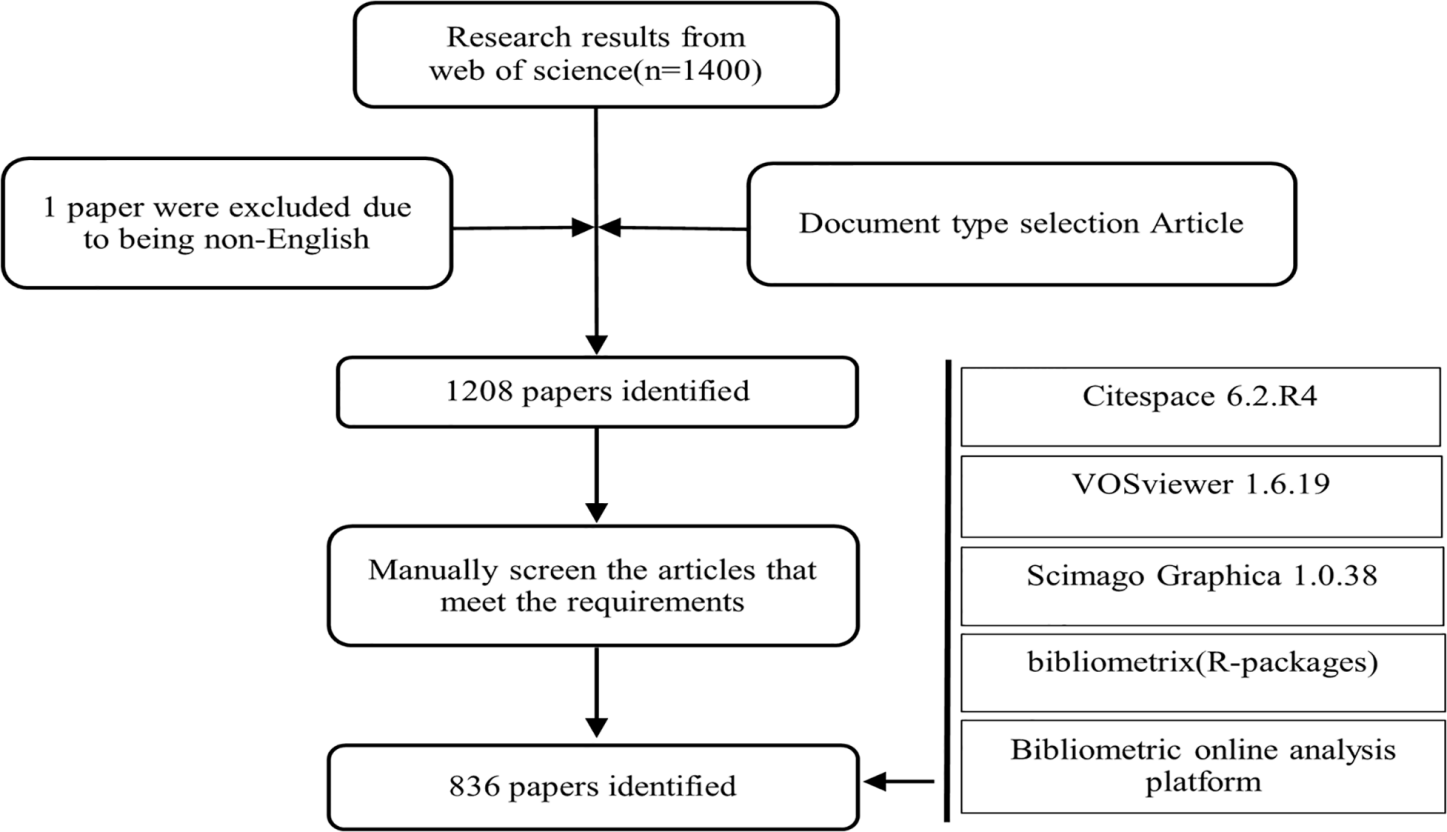
Flowchart of document search, screening, and data analysis.

According to the search strategy, a total of 1400 documents were retrieved; however, after screening according to the inclusion and exclusion criteria, 836 documents were used for further analysis (**Figure 1**).

The data visualization software tools used were CiteSpace 6.2.R4, VOSviewer 1.6.19, Scimago Graphica 1.0.38, Pajek64 5.18, a bibliometric online analysis platform, and Bibliometrix (an R programming language package). CiteSpace is an interactive visual analysis software developed by Professor Chen Chaomei that can carry out different types of network analysis, such as keyword network, state-institution cooperation network, author cooperation network, co-citation periodical cooperation network, and keyword co-occurrence analysis. It is helpful for intuitively analyzing knowledge fields and emerging trends (13, 14). The VOSviewer software was developed by Van Eck and Waltman at Leiden University in the Netherlands in 2009 and is suitable for visualizing data analysis and constructing complex networks of large-scale data (15). Scimago Graphica provides a new way to explore, visually communicate, and understand data. It creates a global map and shows the number of papers published by countries worldwide. Bibliometrix supports the recommended workflow for performing bibliometric analysis. As it is programmed in R, the proposed tool is flexible and can be rapidly upgraded and integrated with other statistical R-packages. It is therefore useful in a constantly changing science such as bibliometrics (16).

## 3 Results

### 3.1 An overview of the annual growth trend

**Figure 2:**
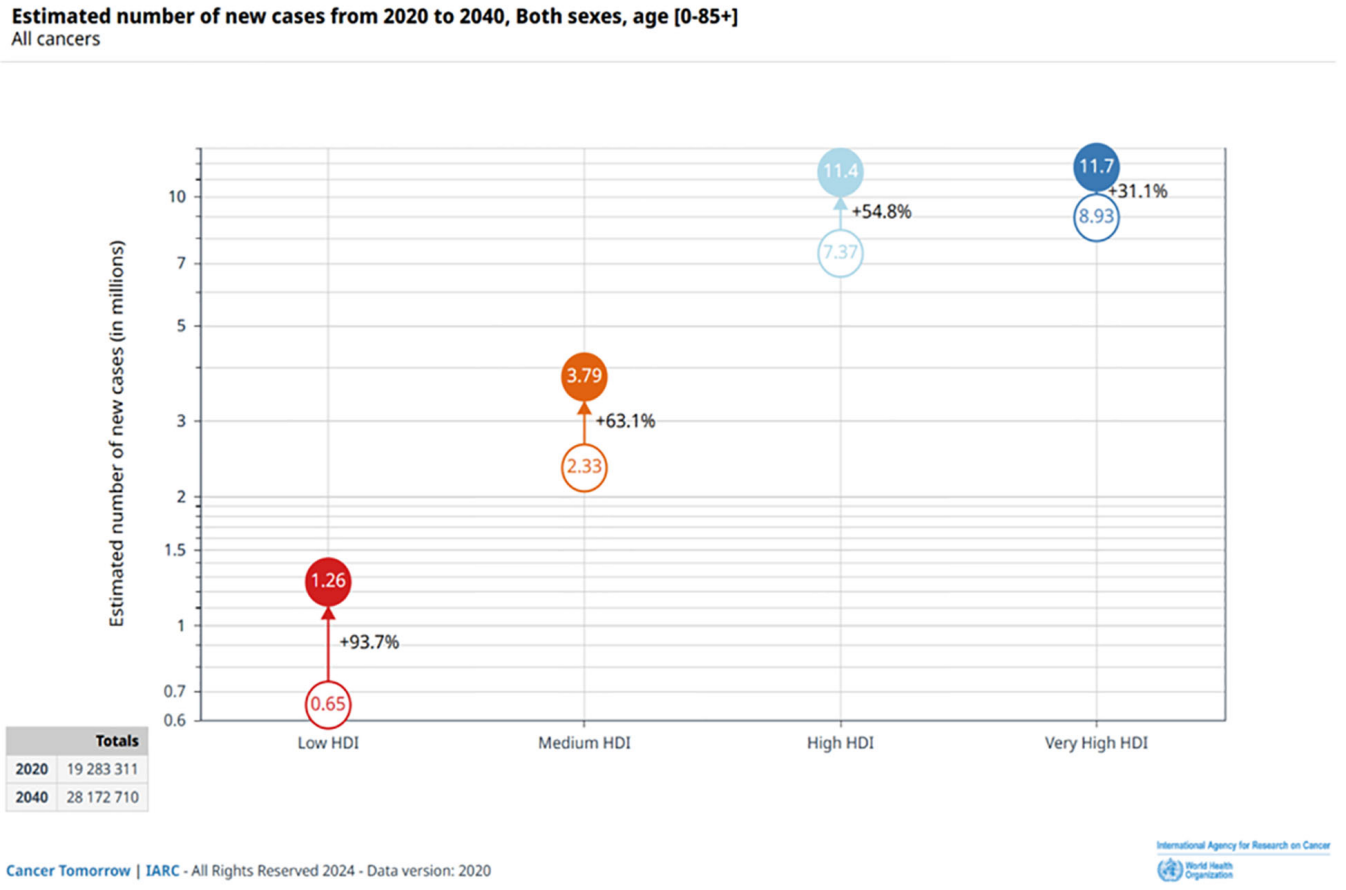
Estimated number of new cases from 2020 to 2040, Both Sexes Combined, age [0-85+], According to the 4-Tier Human Development Index (HDI). Source: https://gco.iarc.fr/.

According to cancer data released by GLOBOCAN 2020, there would be nearly 19.3 million new cancer cases and nearly 10 million new cancer deaths globally by 2020, and GLOBOCAN’s 2020 cancer data predicts that there will be 28 million new cancer cases globally by 2040 (17) (**Figure 2**).

**Figure 3:**
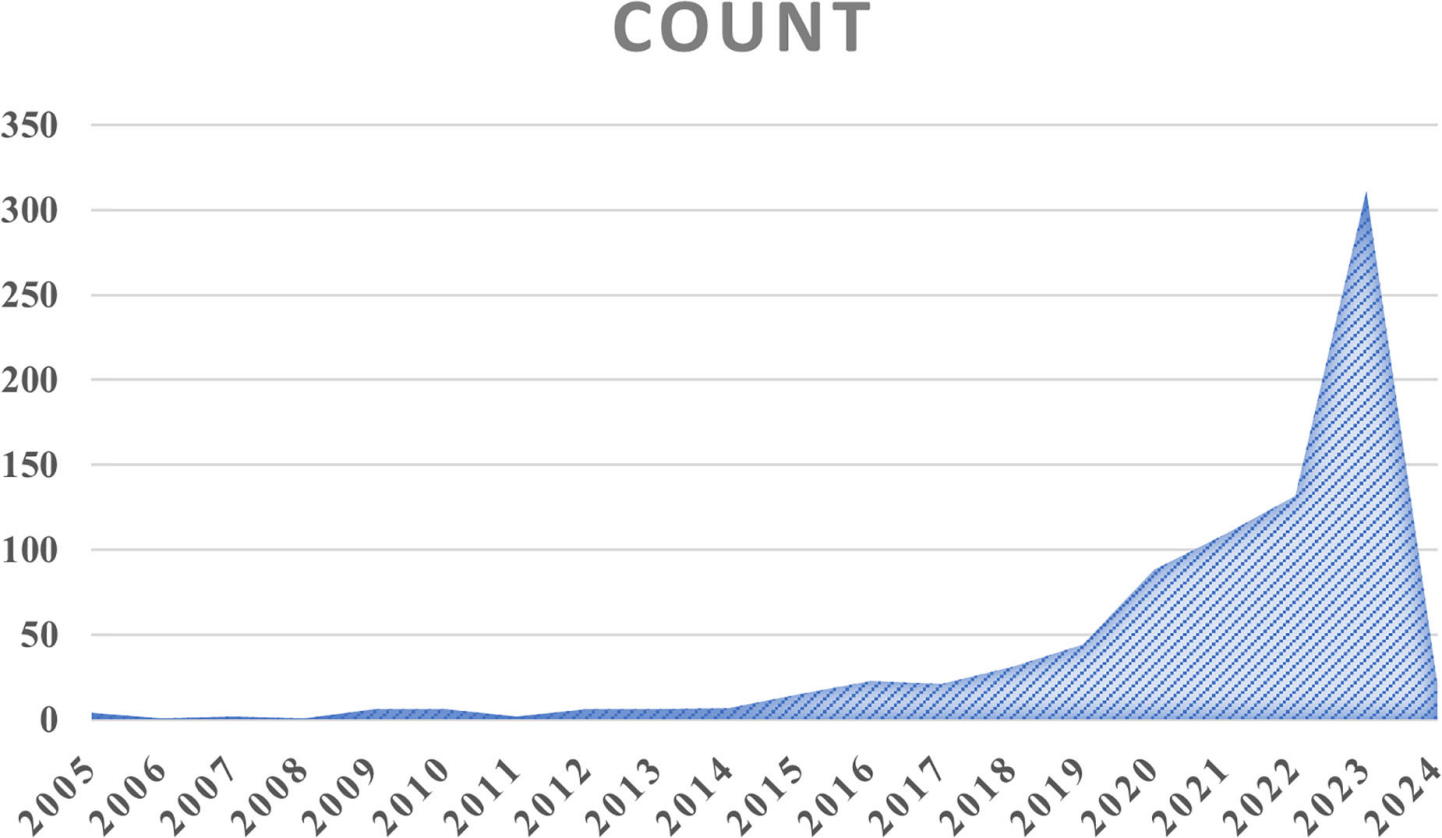
Annual publication volume.

The data were processed using Bibliometrix R-package, and relevant information about the literature was obtained. According to the literature data, the earliest study on the incidence of cancer using the Mendelian randomization approach was published in 2005. As of January 31, 2024, 836 articles have been published (**Figure 3**).

As of January 31, 2024, the annual growth rate of Mendelian randomization and cancer-related articles is 9.12%. These articles were obtained from 243 journals and magazines, and each article cites 15.29 references (**Table 1**); a total of 24369 references were cited. Starting in 2017, the number of publications exploded until reaching a peak in 2023.

**Table 1:** Mendelian Randomized-Cancer Literature data.

Description	Results
Timespan	2005:2024
Sources (Journals, Books, etc)	243
Documents	836
Annual Growth Rate %	9.12
Document Average Age	3.2
Average citations per doc	15.29
References	24369

### 3.2 Distribution of countries or regions and institutions

**Figure 4:**
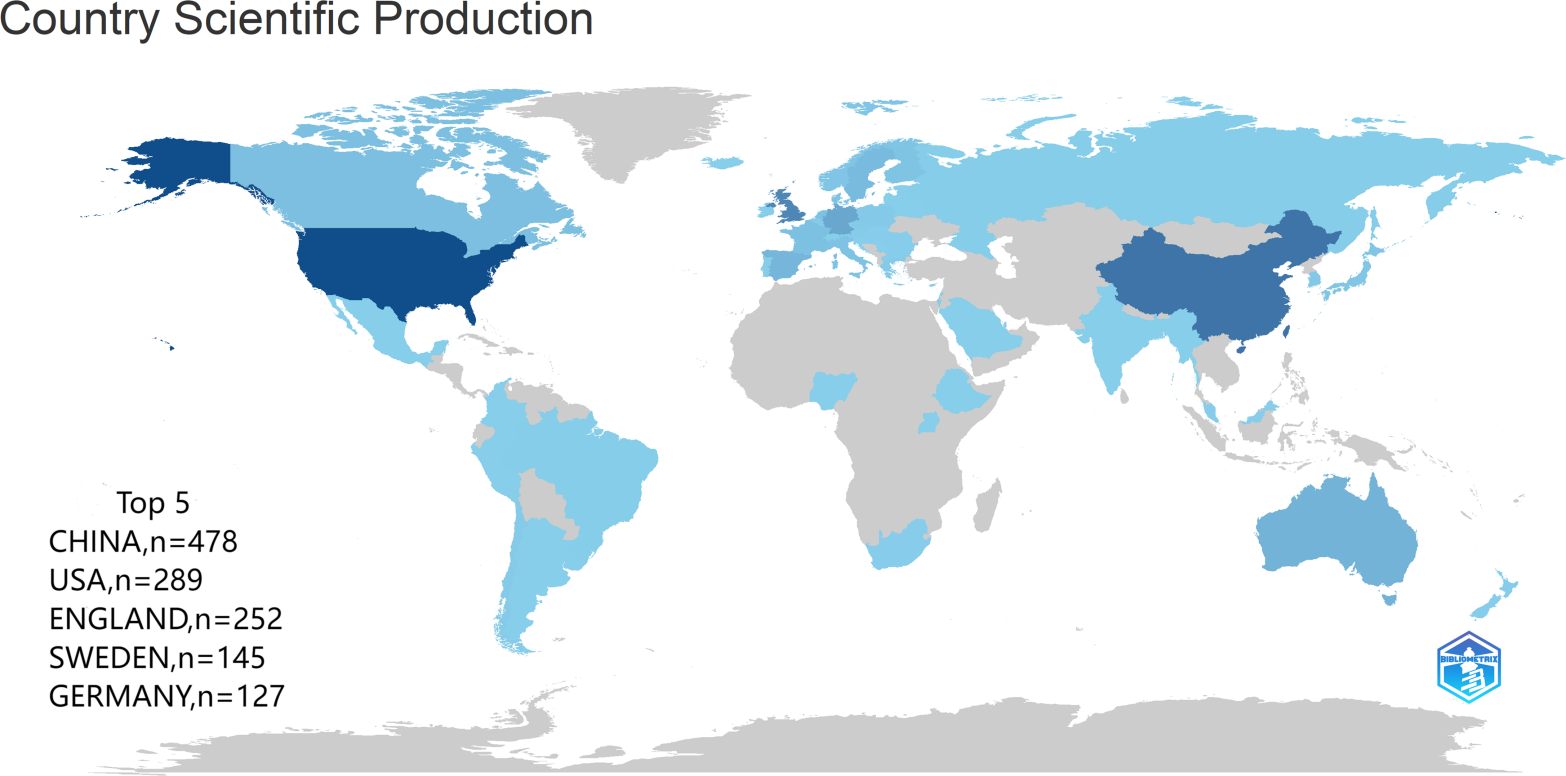
Map of the world (number of articles published by country).

The 836 articles selected in this study came from 72 different countries or regions (**Figure 4**), of which the top 10 were CHINA (n=478), USA (n=289), ENGLAND (n=313), SWEDEN (n=145), GERMANY (n=127), AUSTRALIA (n=122), FRANCE (n=108), CANADA (n=88), DENMARK (n=79), and SPAIN (n=78). The number of articles jointly published by countries in cooperation with each other is shown in **Table 2**.

**Table 2:** Countries with the number of published articles in the top 10.

Rank	Count	Centrality	Year	Countries
1	478	0	2015	CHINA
2	289	0	2008	USA
3	252	0.06	2005	ENGLAND
4	145	0.02	2007	SWEDEN
5	127	0.26	2005	GERMANY
6	122	0.06	2012	AUSTRALIA
7	108	0.22	2005	FRANCE
8	88	0.23	2009	CANADA
9	79	0.1	2007	DENMARK
10	478	0	2015	PEOPLES R CHINA

**Figure 5:**
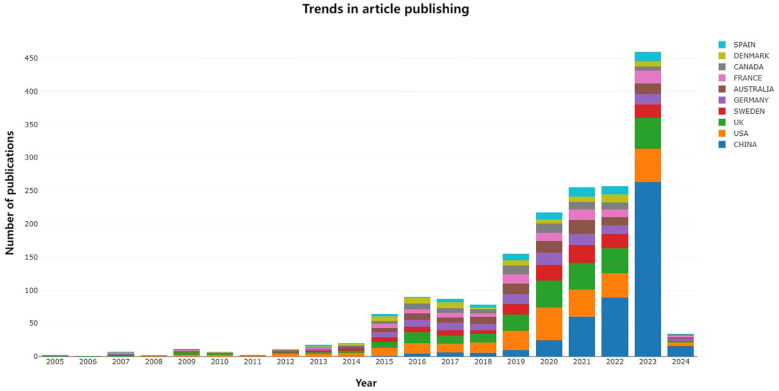
Bar chart of the top ten countries in the number of articles published.

**Figure 6:**
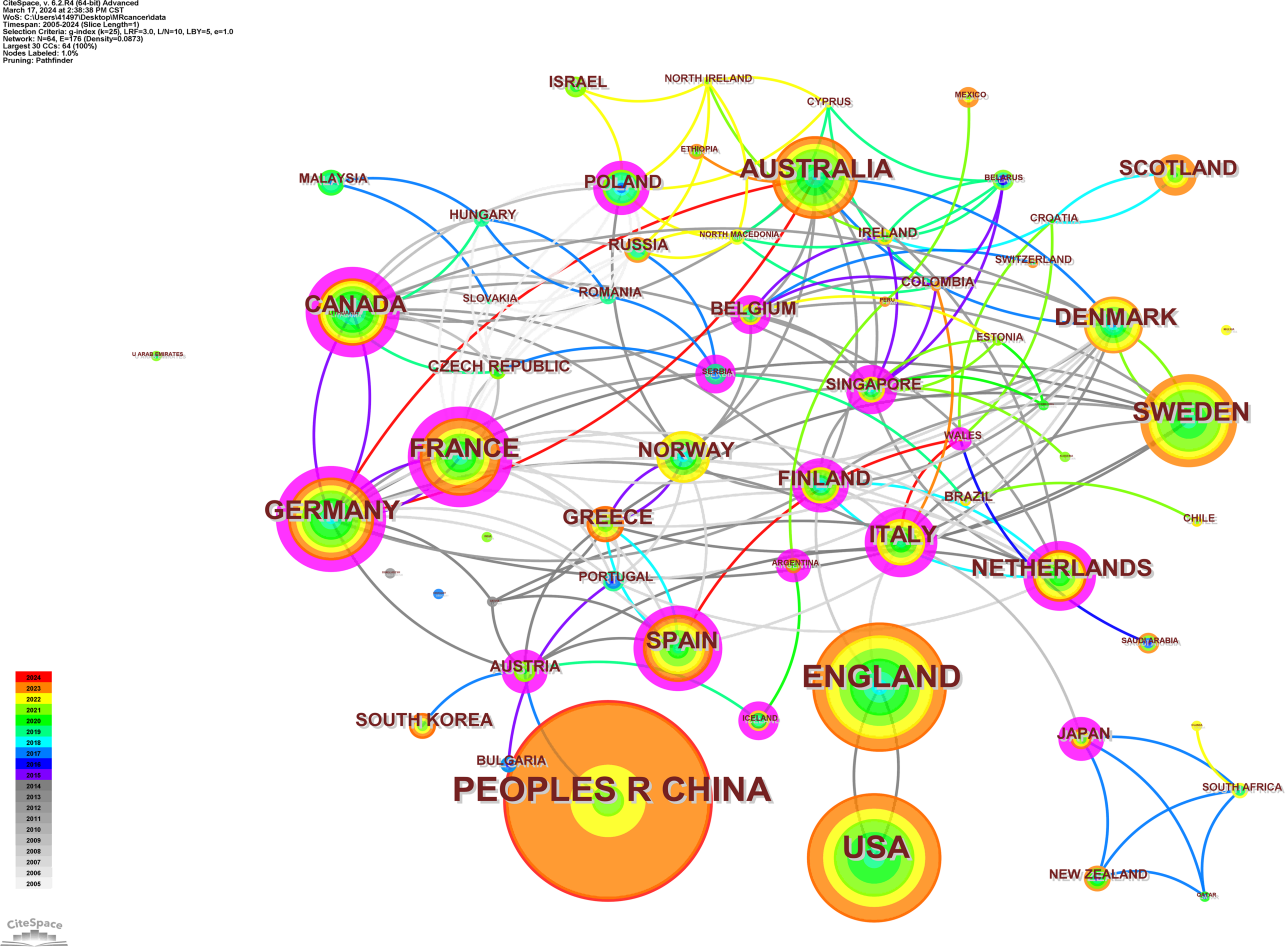
The country cooperation map.

Germany and the UK were the first countries to publish Mendelian randomization-cancer articles. The number of articles from the United States has increased steadily since 2015, indicating that research in this field has tended to be stable. The first Chinese article on the application of Mendelian randomization in the field of cancer was published in 2015, and the number of articles published has increased since 2019, indicating that an increasing number of Chinese researchers are beginning to explore this field (**Figure 5**). The country cooperation map shows the academic cooperation between various countries and regions. Italy has the most extensive cooperation with other countries. Among the top 10 countries in terms of the number of articles published, Germany has the most extensive academic cooperation with other countries, and there is also close cooperation among European countries. In addition, communication between other countries and regions needs to be strengthened (**Figure 6**).

**Figure 7:**
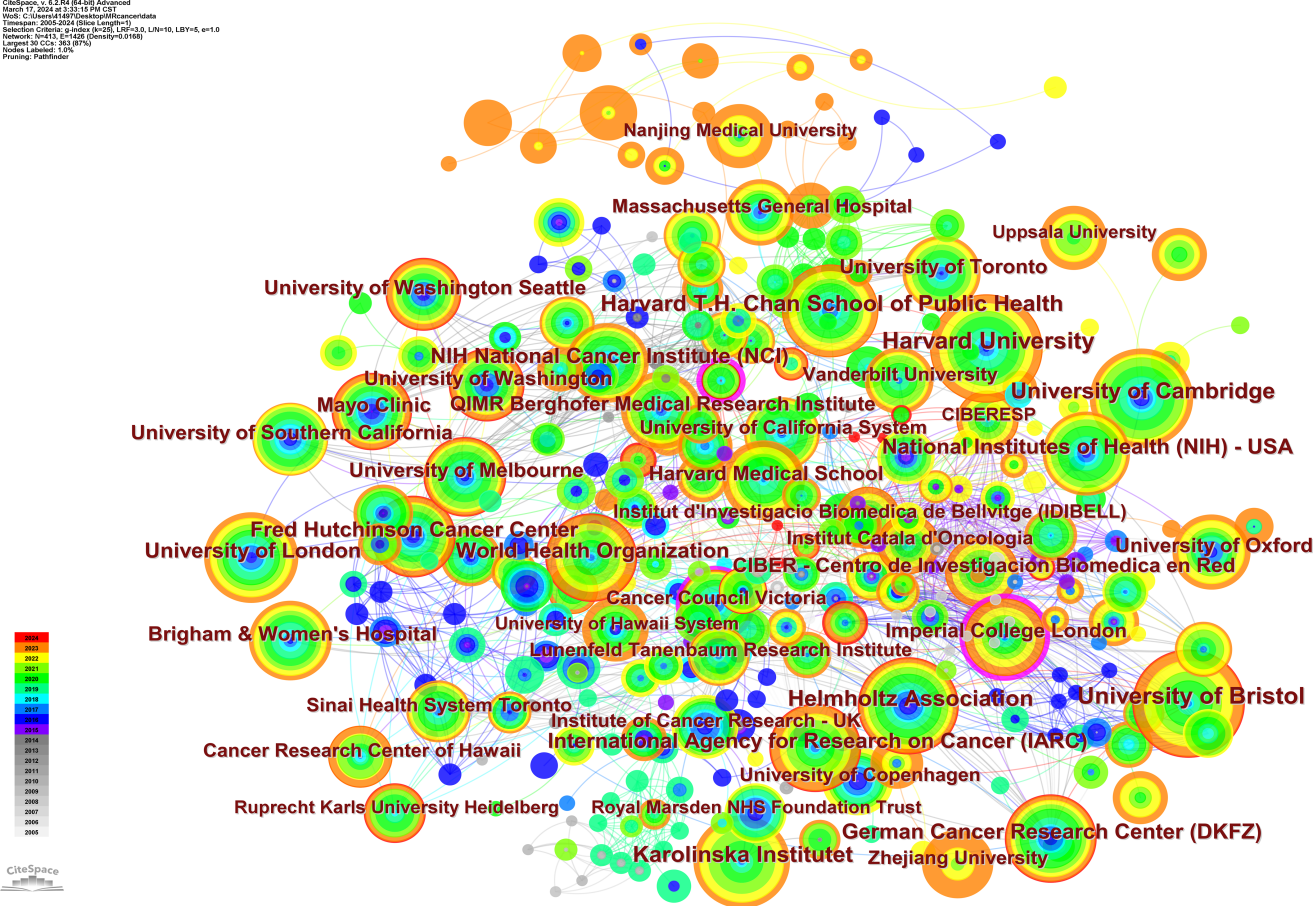
Institutional co-occurrence map.

As shown in **Figure 7**, each node represents an organization. The node size represents the number of articles published by the organization, internal color of the node represents a different publication time, and connection between nodes represents cooperation between the organizations. The thicker the line, the closer the cooperation between the agencies. A total of 413 institutions worldwide have published articles in this field. The top 10 institutions in terms of the number of published papers were mainly from the United Kingdom (n=3), the United States (n=2), Germany (n=2), Sweden (n= 1), Switzerland (n=1), and France (n=1) (**Table 3**). From the centrality value in **Table 3**, we can see that among the top 10 institutions in terms of the number of articles published, the Helmholtz Association and German Cancer Research Center (DKFZ) cooperated most extensively with other academic institutions, whereas the remaining eight institutions preferred to conduct research independently. The central value is used to describe the degree of cooperation between organizations. Of the 417 institutions, the top three institutions with the most extensive cooperation are Imperial College London (Centrality=0.25), Kaiser Permanente (Centrality=0.23), and Cancer Council Victoria (Centrality=0.17). (A centrality value of 0 does not mean that there has never been any cooperation with any other organization, but that the amount of cooperation is smaller than other institutions.).

**Table 3:** The top ten institutions in the number of articles published.

Rank	Count	Centrality	Year	Institutions
1	130	0.05	2012	Harvard University
2	123	0	2005	University of Bristol
3	103	0.01	2009	Karolinska Institutet
4	97	0.01	2013	University of Cambridge
5	95	0.08	2007	Helmholtz Association
6	92	0.02	2012	Harvard T.H. Chan School of Public Health
7	88	0.06	2007	German Cancer Research Center (DKFZ)
8	87	0.04	2005	International Agency for Research on Cancer (IARC)
9	83	0.01	2005	World Health Organization
10	79	0	2015	University of London

### 3.3 Distribution and co-authorship of authors

A total of 643 authors worldwide have published articles in the field of Mendelian randomization-cancer. The top ten authors of published articles were from the UK (n=3), Germany (n=2), Australia (n=1), China (n=1), the USA (n=1), France (n=1), and Sweden (n=1) (**Table 4**). Smith, George Davey—a professor of the University of Bristol—is an expert in many fields, including Environmental & Occupational Health, General & Internal Medicine, Genetics & Heredity, and Cardiovascular System & Cardiology. He specializes in Mendelian randomization research methods for studying disease pathogenesis. One of the most cited articles on Mendelian randomization published by the professor discusses how to properly use data from GWAS and proposes a validation method that provides a sensitivity analysis for the robustness of the findings from a Mendelian randomization investigation (18). He also called on researchers around the world to use the Mendelian method of randomization correctly and carefully, and to apply appropriate statistical methods when screening for “instrumental variables” related to “exposure factors” in order to avoid false-positive results (19).

**Table 4:** The top ten authors of articles published.

Rank	Count	Year	Authors	Country
1	55	2016	Martin, Richard M	UK
2	51	2006	Smith, George Davey	UK
3	43	2016	Zheng, Wei	China
4	42	2015	Brenner, Hermann	Germany
5	42	2015	Giles, Graham G	Australia
6	40	2009	Gunter, Marc J	France
7	36	2015	Le marchand, Loic	USA
8	34	2015	Burgess, Stephen	UK
9	34	2020	Larsson, Susanna C	Sweden
10	33	2015	Chang-claude, Jenny	Germany

He has combined various analytical methods with epidemiology to analyze the pathogenesis of many diseases, making a significant contribution to the global public health field. Martin, Richard M—also from the University of Bristol—is an expert in many fields, including Oncology, General & Internal Medicine, and Nutrition & Dietetics. One of the most cited articles in the field of Mendelian randomization-cancer concerns a study of the causal relationship between telomere length and the incidence of cancer and non-oncologic disease, and the results showed that telomere length was positively associated with cancer risk (20). Professor Smith, George Davey also participated in this study. These two experts are the core authors in the field of Mendelian Randomization – Cancer.

**Figure 8:**
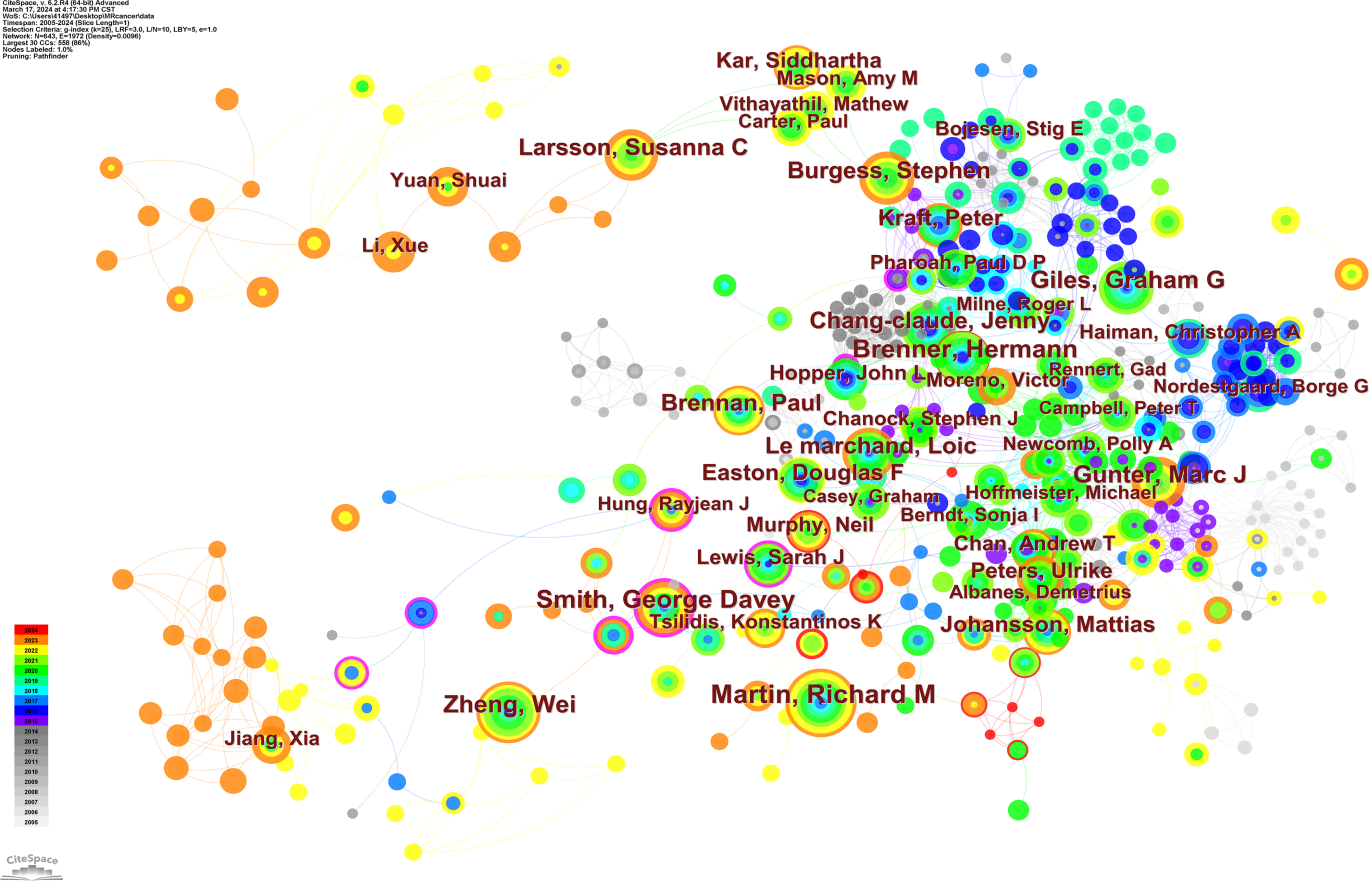
Co-author co-occurrence map.

The co-author co-occurrence map visually shows the cooperation of authors. Each node represents an author, the node size represents the number of articles published, color within the node represents the publication time of the article, and the more the line segments between the nodes, the more extensive the cooperation between the authors (**Figure 8**). The authors with the most extensive collaborations were Haycock, Philip C (n=11, Centrality=0.15); Smith, George Davey (n=51, Centrality=0.14); and Lewis, Sarah J (n=19, Centrality=0.13).

### 3.4 Analysis of journals and co-cited academic journals

**Figure 9:**
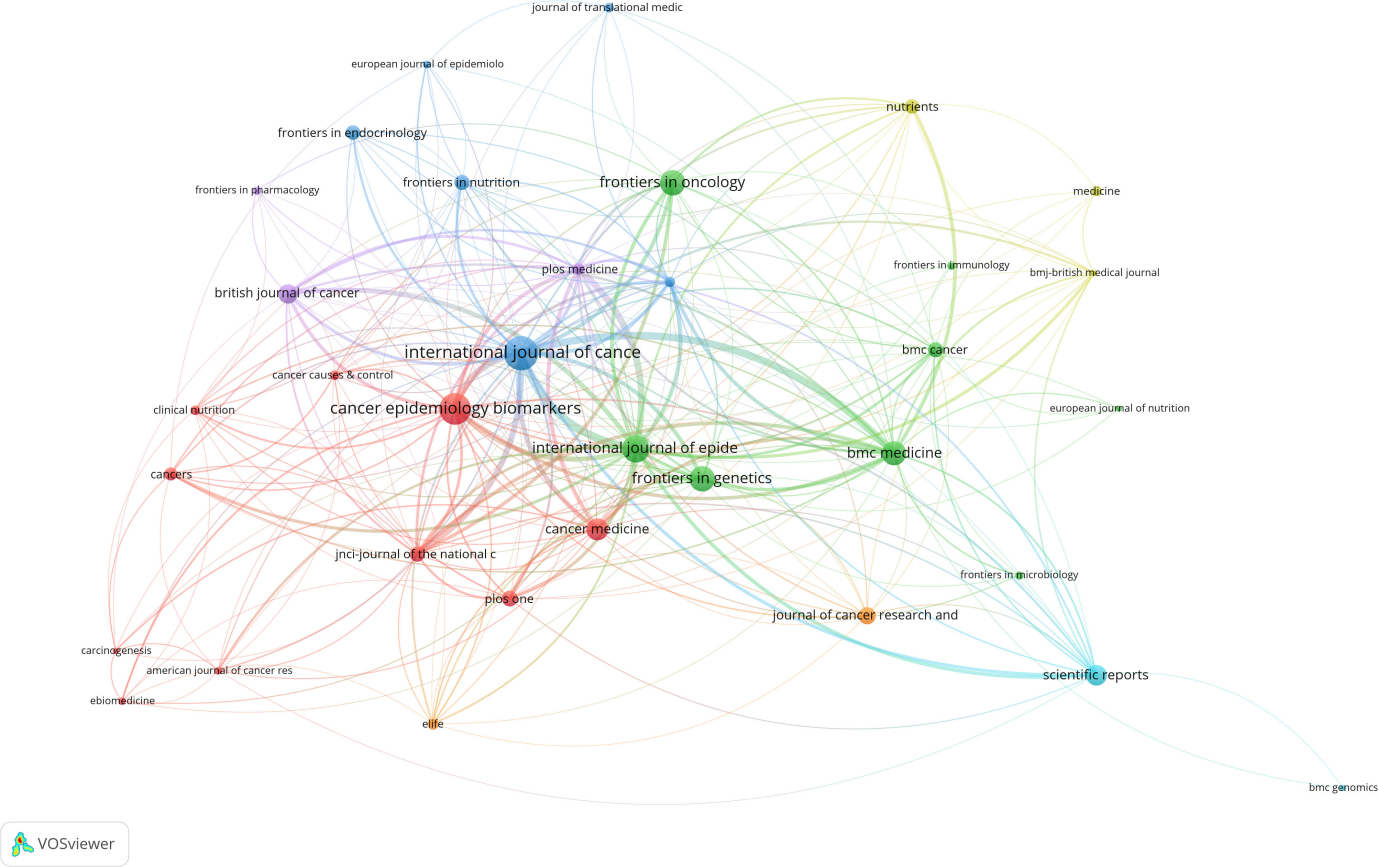
Journal cluster map.

This study included 836 articles published in 243 journals. Among the top ten journals with the highest number of published articles in this field, six belong to the field of oncology (International Journal of Cancer, Cancer Epidemiology Biomarkers & Prevention, Frontiers in Oncology, Cancer Medicine, Journal of Cancer Research and Clinical Oncology, and British Journal of Cancer), two belong to comprehensive journals (Scientific Reports and BMC Medicine), and the other issues are related to epidemiology and public health (**Table 5**). After clustering with the VOSviewer software, there were five clusters in total. As shown in **Table 5**, each node represents a journal, node size represents the number of articles, line segment represents the association strength between journals, and different colors represent different clusters. The journals in red clustering are mainly related to cancer, journals in green clustering are mainly related to genetics and immunology, and blue clustering and other color clustering belong to comprehensive medical or comprehensive journals (**Figure 9**).

**Table 5:** Top 10 journals with the largest number of articles in this field.

Sources	Articles	IF (2022)	Category Quartile
INTERNATIONAL JOURNAL OF CANCER	45	6.4	Q1
CANCER EPIDEMIOLOGY BIOMARKERS & PREVENTION	40	3.8	Q2
INTERNATIONAL JOURNAL OF EPIDEMIOLOGY	33	7.7	Q1
FRONTIERS IN GENETICS	30	3.7	Q2
FRONTIERS IN ONCOLOGY	30	4.7	Q2
BMC MEDICINE	28	9.3	Q1
CANCER MEDICINE	25	4	Q2
SCIENTIFIC REPORTS	23	4.6	Q2
BRITISH JOURNAL OF CANCER	21	8.8	Q1
JOURNAL OF CANCER RESEARCH AND CLINICAL ONCOLOGY	18	3.6	Q2

The top three most cited journals were the International Journal of Epidemiology (IF 2022, 7.7; 718 citations), Nature Genetics (IF 2022, 30.8; 688 citations), and the International Journal of Cancer (IF 2022, 6.4; 517 citations) (**Table 6**).

**Table 6:** The top ten journals with the highest number of cited journals.

Count	Centrality	Year	Cited Journals	IF (2022)	Category Quartile
718	0.03	2005	INT J EPIDEMIOL	7.7	Q1
688	0.01	2008	NAT GENET	30.8	Q1
517	0.02	2005	INT J CANCER	6.4	Q1
487	0.01	2009	GENET EPIDEMIOL	2.1	Q3
440	0.01	2010	NATURE	64.8	Q1
430	0.06	2005	CANCER EPIDEM BIOMAR	3.8	Q2
395	0.03	2008	HUM MOL GENET	3.5	Q2
388	0	2015	NAT COMMUN	16.6	Q1
372	0.11	2006	AM J EPIDEMIOL	5	Q1
354	0	2012	PLOS ONE	3.7	Q2

**Figure 10:**
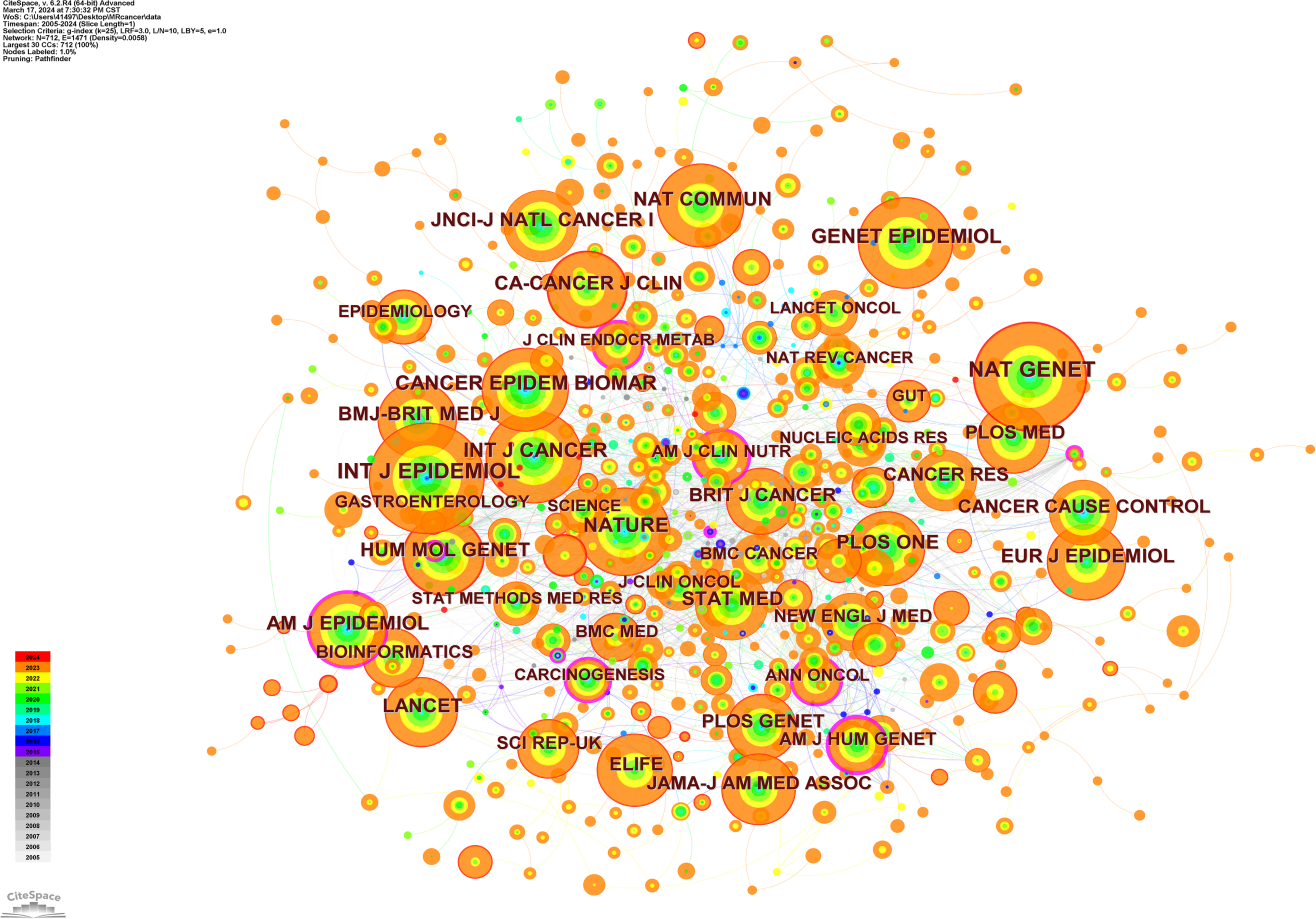
Co-occurrence map of cited journals.

The co-occurrence map of the cited journals shows that three journals, Annals of Human Genetics (centrality=0.31), American Journal of Human Genetics (centrality=0.26), and American Journal of Clinical Nutrition (centrality=0.19), have the highest centrality values according to the numerical ranking of centrality, indicating that these three journals are cited along with a large number of other journals (**Figure 10**). The above impact factor values were derived from Web of Science data.

### 3.5 Keywords analysis

From the 836 articles included in this study, 546 keywords were used. **Table 7** shows the top ten keywords in terms of frequency (excluding the keywords “Mendelian randomization” and “cancer”). We sorted all the keywords related to the types of cancer, and the top four with the highest frequency were “breast cancer”, “colorectal cancer”, “prostate cancer”, and “lung cancer”. “Breast cancer” and “colorectal cancer” were the earliest to be combined with Mendelian randomization research methods. The combination of research on “thyroid cancer,” “liver cancer,” “oropharyngeal cancer,” and “nonmelanoma skin cancer” with Mendelian randomization methods was relatively late, in 2023 (**Table 8**).

**Table 7:** The top ten keywords in frequency.

Count	Centrality	Year	Keywords
227	0	2005	risk
182	0.29	2005	association
154	0	2010	meta analysis
153	0.04	2016	instruments
132	0.01	2010	genome wide association
125	0.2	2009	breast cancer
100	0.19	2009	colorectal cancer
100	0.04	2015	variants
97	0	2018	bias
97	0.04	2009	epidemiology

**Table 8:** The types of cancer in key words.

Count	Centrality	Year	Types of cancer
125	0.2	2009	breast cancer
100	0.19	2009	colorectal cancer
94	0.06	2009	lung cancer
78	0.02	2015	prostate cancer
21	0.01	2019	ovarian cancer
19	0.05	2017	pancreatic cancer
16	0	2018	bladder cancer
16	0.02	2019	endometrial cancer
13	0.23	2005	colon cancer
11	0.08	2021	renal cell carcinoma
10	0.02	2014	esophageal cancer
10	0.02	2012	gastric cancer
9	0	2023	thyroid cancer
6	0	2020	skin cancer
5	0.01	2016	cervical cancer
5	0.03	2007	adenocarcinoma
5	0.02	2020	squamous cell carcinoma
7	0	2023	liver cancer
4	0.01	2023	oropharyngeal cancer
4	0	2023	nonmelanoma skin cancer
3	0	2023	testicular cancer
2	0.01	2007	cardia cancer
2	0	2022	kidney cancer
2	0	2022	esophageal adenocarcinoma
2	0	2021	epithelial ovarian cancer
2	0.03	2016	rectal cancer
2	0	2020	neck cancer

**Figure 11:**
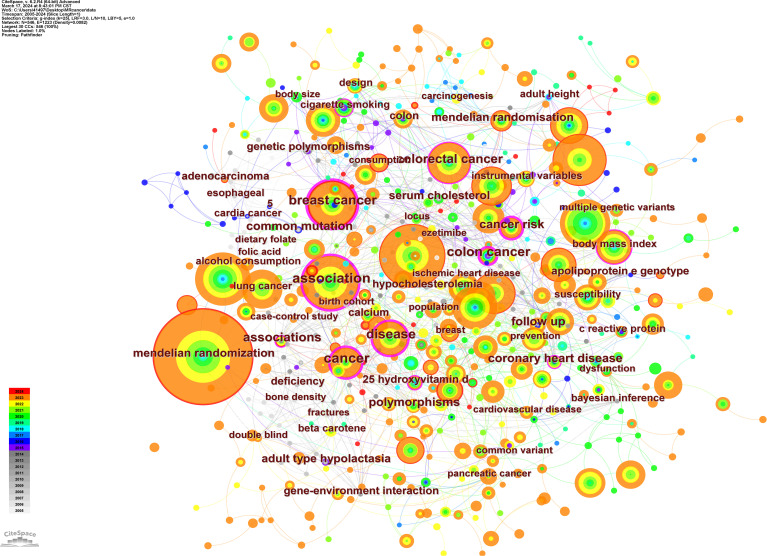
Keyword co-occurrence map.

The keyword co-occurrence map shows the relationship between keywords and that between keywords and time. Each node represents a keyword; the color of the dot represents the time when the keyword appears, and line segment represents the correlation between keywords (**Figure 11**). The 15 terms with the highest epidemic intensity are listed in **Table 9**. From the Keywords with the Strongest Citation Bursts table, the keyword “cigarette smoking” has been consistently high since it appeared in 2012. From 2012 to 2021, a total of 16 papers reported on the relationship between smoking and cancer risk, among which the most cited paper was published by Larsson SC, Carter P, Kar S, et al., which discussed whether long-term smoking and alcohol consumption would increase cancer risk. The results showed that smoking may be a risk factor for head and neck, esophageal, stomach, cervical, and bladder cancer (21).

**Table 9:** Top 15 Keywords with the strongest citation bursts.

Keywords	Year	Strength	Begin	End	2005 - 2023
cigarette smoking	2012	3.78	2012	2021	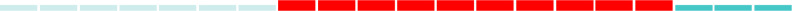
cancer risk	2012	3.68	2012	2019	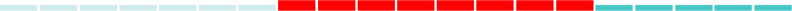
body mass index	2009	5.92	2014	2018	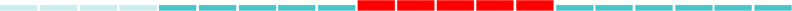
instrumental variables	2014	3.14	2014	2018	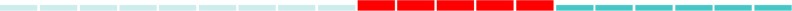
genome wide association	2010	13.97	2015	2020	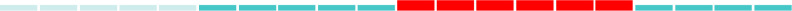
prostate cancer	2015	5.04	2015	2017	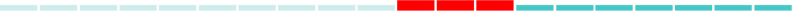
susceptibility loci	2015	4.69	2015	2019	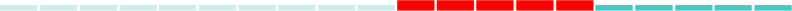
metaanalysis	2010	9.24	2016	2018	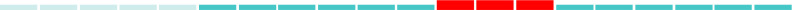
mendelian randomization	2016	3.15	2016	2018	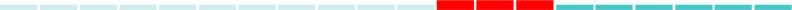
vitamin d	2018	4.99	2018	2020	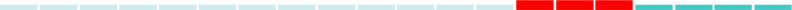
smoking	2018	3.92	2018	2019	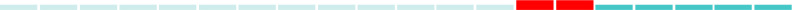
insulin	2018	3.4	2018	2021	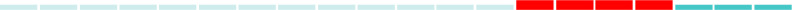
identification	2019	3.29	2019	2022	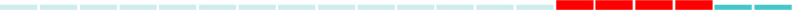
pleiotropic genetic variants	2019	3.21	2019	2020	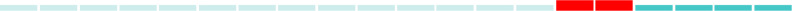
prevention	2018	3.52	2021	2022	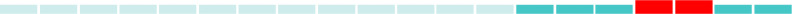

### 3.6 MR data source

We analyzed the commonly used MR analysis databases in the included articles. Some of the GWAS data in these MR studies were sourced from relevant GWAS meta-analysis publications, while others were obtained from specialized fields (such as psychiatric GWAS databases) or comprehensive GWAS databases. The UK Biobank is one of the most commonly used GWAS databases in MR research (22). This is an open-source GWAS comprehensive database that collects genetic phenotypes including but not limited to BMI, cardiovascular disease, cancer, diet, and daily behavior. The UK Biobank database collected 7,221 phenotypes from 6 continental ancestral populations for GWAS analysis (**Table 10**), completed by Neale laboratory (23). The FinnGen database was initiated in Finland in 2017 and encompasses genetic data and health registry data from 500,000 Finns, inclusive of GWAS data for 1,932 diseases (24). The Psychiatric Genomics Consortium (PGC) database is a repository of data and resources dedicated to advancing the understanding of the genetic and molecular basis of psychiatric disorders. Its scope encompasses a range of research areas, including genome-wide association studies (GWAS) and behavioral investigations into the etiology and pathogenesis of bipolar disorder, Alzheimer’s disease, autism, attention deficit hyperactivity disorder (ADHD), and anxiety (25). CKDGen Consortium is an open-source GWAS-Meta database that collects and publishes GWAS-Meta analyses on kidney function diseases (26). The Early Growth Genetics (EGG) consortium is dedicated to the analysis of GWAS data spanning the fetus to young adult period. This encompasses a range of data types, including fetal data, maternal data, birth weight, childhood BMI, and combined data from adolescence (27). The Social Science Genetic Association Consortium (SSGAC) is a database of genetic association studies related to social sciences. The database is a repository of data that can be queried to identify correlations between genetic variants and social phenomena (28).

**Table 10:** UK Biobank population phenotyping data.

Population	Num. Individuals	Num. Phenotypes
African ancestry	6636	2493
Admixed American ancestry	980	1105
Central/South Asian ancestry	8876	2771
East Asian ancestry	2709	1612
European ancestry	420531	7200
Middle Eastern ancestry	1599	1372

## 4 Discussion

**Figure 12:**
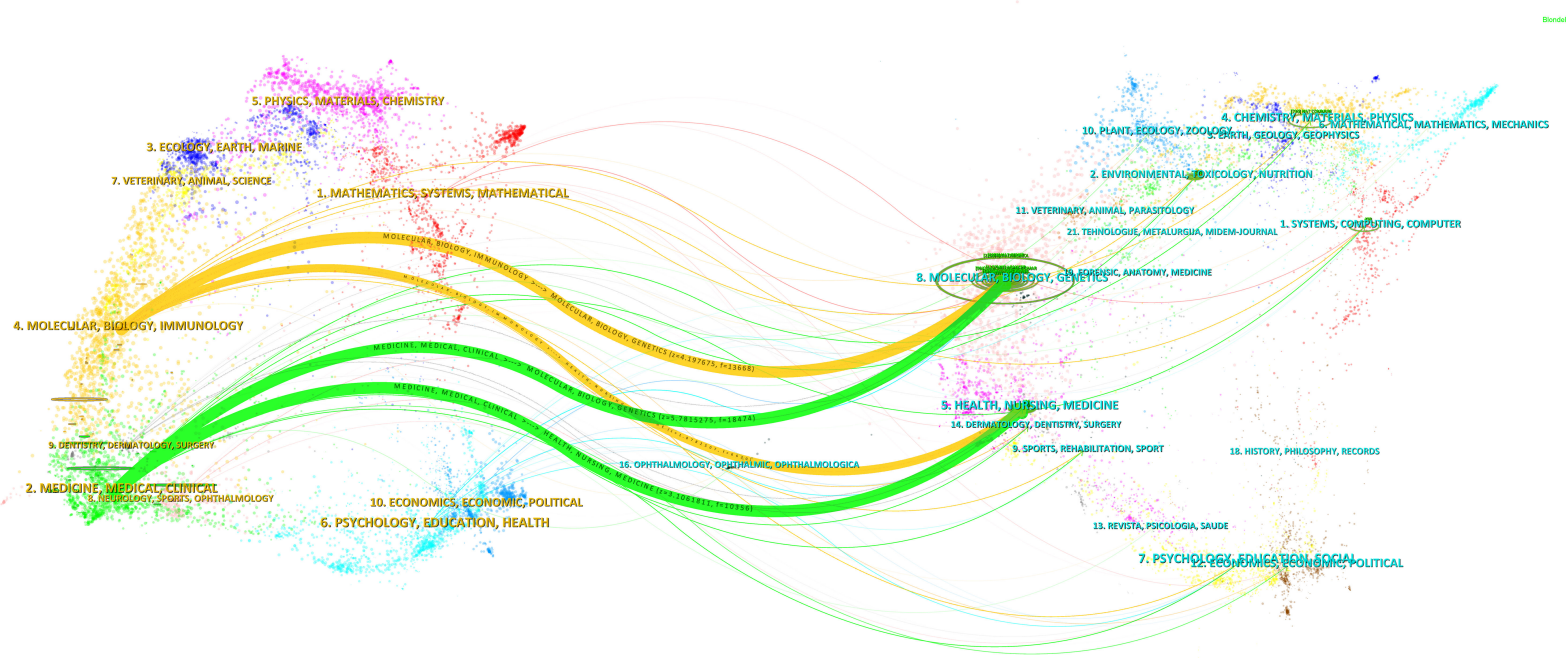
The dual journal map overlay.

The dual journal map overlay reveals two main citation pathways (produced by the CiteSpace software) (29): clustering of journals published within the field on the left (cutting-edge knowledge) and clustering of cited journals on the right (basic knowledge). Articles related to the Mendelian randomization-cancer field are published in two main clustering domains: the first clustering domain includes Molecular Biology and Genetics, and the second domain mainly includes Health, Nursing, and Medicine. The two main relevant clustering domains within the field are derived from the two main clustering domains of the journals of the cited literature: the first one mainly includes Molecular Biology and Immunology, and the second category mainly includes Medicine, Medical, and Clinical journals. **Figure 12** shows the dual journal map overlay visualization.

The Mendelian randomization research method is based on genetics and epidemiology and derives causality from genetic data according to the Mendelian laws of genetics (30). As GWAS become more and more in-depth, the “instrumental variables” available to researchers in Mendelian randomization studies are becoming increasingly accurate, which is simultaneously more conducive to the diversity of “exposure factor” choices and the breadth and accuracy of “confounding factor” exclusions (31, 32). The continuous improvement of GWAS databases not only promotes the study of cancer pathogenesis but also plays a key role in the exploration of prognostic markers for cancer (33, 34). Open-source data analysis platforms based on GWAS databases also play an important role (35).

Our bibliometric analysis revealed that the most frequently utilized GWAS database is the UK Biobank. Although the database contains GWAS data on 7,221 phenotypes from six continental ancestral groups, its primary focus is on European populations. In addition, since population weights are rarely mentioned in Mendelian randomization studies, the UK Biobank database alone cannot represent all global populations. Our findings revealed a paucity of GWAS studies on Asian populations, resulting in a dearth of two-sample Mendelian randomization studies targeting Asian populations or genetic phenotypes. It remains unclear whether these Mendelian randomization results are biased by race.

We counted all the types of cancer in the keywords of 836 articles, and the most frequently occurring keyword was breast cancer, and there was a wealth of research on the “exposure factors” that contribute to the risk of breast cancer. A higher relative sugar intake can genetically increase the risk of Luminal B and HER2-positive breast cancer (36). There is also an association between the gut flora microbes and breast cancer, with studies showing that an increased abundance of the Genus_Sellimonas is causally associated with an increased risk of ER+ breast cancer (37). In terms of diet, the consumption of dried fruits and oily fish may have a protective effect against breast cancer (38), and the intake of vitamin E may reduce the risk of breast cancer (39). However, further evidence is required. In terms of keywords related to cancer types, thyroid cancer (first article published in 2023 and nine related articles in that year) and liver cancer (first article published in 2023 and seven related articles in that year) are likely to become the next research hotspots.

In cancer research, the more common “exposure factors” are generally related to physical signs and behaviors. Through Mendelian randomization, increased smoking and drinking behaviors have been shown to increase the risk of gastroesophageal reflux disease (GERD), which is associated with an increased risk of lung cancer (40). A Mendelian randomization experiment with six sets of “instrumental variables” and 78 associated SNPs obtained from the GWAS database concluded that smoking frequency was significantly and positively associated with bladder cancer risk (41). A two-sample Mendelian randomization study demonstrated that insomnia was positively associated with the risk of lung cancer and that sleep duration played a protective role in lung cancer (42). Coffee consumption is positively associated with the risk of digestive system cancers, especially esophageal cancer. Coffee consumption was also positively associated with the risk of multiple myeloma (43). The risk of colorectal cancer in men was associated with a high body mass index (BMI), although its association in women remained unclear. Carrying more alleles for BMI is associated with a higher risk of colorectal cancer (44). In addition, there are some unconventional “exposure factors.” Several factors, including income, education, BMI, and smoking were causally associated with squamous cell lung cancer and overall lung cancer (45). Smoking and education independently correlated with overall lung cancer and squamous cell lung cancer (46). A genetic predisposition of 3.6 years of education above the average reduced the risk of developing lung cancer by 52%, and low education was one of the risk factors for the development of lung cancer (47). Similarly, genetic predictions supported the protective effect of higher educational attainment against esophageal cancer and GERD (48).

It is worth noting that changes in mood are an important factor affecting cancer risk. A number of mental illnesses can lead to an increased risk of cancer. Depression can increase the risk of cervical cancer, and bioinformatics studies have shown that these two diseases are highly related to the PI3K-Akt signaling pathway (49). Another Mendelian randomization study demonstrated that depression increases the risk of breast cancer. Depression is considered a chronic stressor, and this long-term stress may lead to immune dysfunction in the body (50). Major depression, schizophrenia, and bipolar affective disorder are all risk factors for thyroid cancer, and in a 5-year follow-up study, patients with major depression had an overall 1.62-fold increased risk of cancer (51).

**Figure 13:**
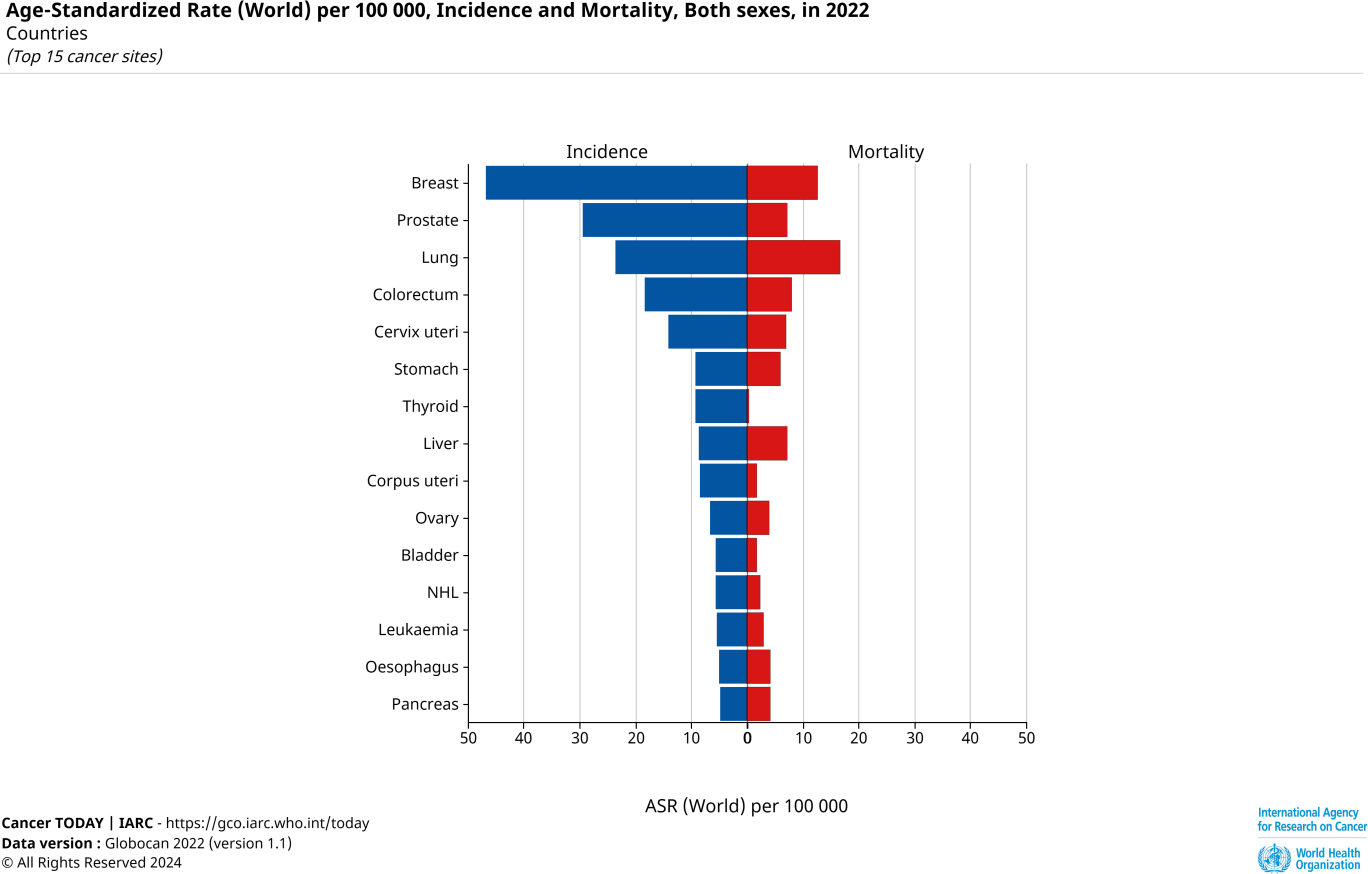
Top 15 cancer incidence rates (per 100,000).

We analyzed the keywords, abstracts, journals, and other relevant information of 836 articles and followed the bibliometric analysis method to compile and organize the data and analyze the hidden relationships between various types of information (52). The bibliometric analysis method can indirectly discover current research hotspots in the field of Mendelian randomization-cancer, and simultaneously predict the trends of future research hotspots within this field (53). We inferred the following conclusions from the keywords related to cancer types: articles related to cancers other than breast, colorectal, lung, and prostate cancers will increase in the future. Among them, the fastest-growing ones may be uterine, renal cell, and thyroid cancers, because articles related to these three types of cancers appeared later, but the number of articles was relatively high compared to other cancers in the short term. Contrarily, according to the latest data from the International Agency for Research on Cancer (IARC) (the latest statistics are as of 2022), uterine and thyroid cancers are relatively more prevalent globally (**Figure 13**). In terms of the selection of “exposure factors,” when studying the pathogenesis of various cancers, there are relatively more articles on the selection of more conventional and specific “exposure factors,” such as height, weight, body fat percentage, and smoking or drinking habits, while there are fewer studies on the selection of relatively abstract “exposure factors,” such as education level, economic status, and environmental factors. With the increasing availability of “instrumental variables” and the incorporation of more statistical methods, it is likely that there will be more studies on relatively abstract “exposure factors” in the future.

Although an increasing number of researchers worldwide are beginning to use Mendelian randomization methods for more in-depth epidemiological studies, these methods still have some limitations in their current use. For example, when working with genome-wide data, traditional inverse-variance weighted methods can only provide consistent estimates if all genetic variants in the analysis are valid instrumental variables. Depending on the type of data, multiple statistical methods should be applied to ensure the rigor of the research process (54–57). More advanced and practical statistical methods should be used to ensure that the correlation between cause and effect in the content of the study is sufficient to avoid false-positive results.

## 5 Conclusion

This study included a total of 836 articles published by 643 authors in 72 countries and regions. The application of Mendelian randomization in the field of cancer is far from being finished, and there are many directions to be explored in the study of the pathogenesis of various types of cancers. The study of the “exposure factors” will be gradually deepened, and the related research in this field will continue to increase in the next few years, which is a promising research prospect in this field.

## 6 Strengths and limitations

In this study, we have endeavored to elucidate the causal relationship between various exposure factors and cancer through Mendel’s randomization. However, the causal relationship between mental illness and cancer remains inconclusive, and the specific biological mechanism remains elusive.

## Data Availability

The original contributions presented in the study are included in the article/supplementary material. Further inquiries can be directed to the corresponding author.
